# A Centimeter‐Scale Type‐II Weyl Semimetal for Flexible and Fast Ultra‐Broadband Photodetection from Ultraviolet to Sub‐Millimeter Wave Regime

**DOI:** 10.1002/advs.202205609

**Published:** 2023-04-24

**Authors:** Qi Yang, Ximiao Wang, Zhihao He, Yijun Chen, Shuwei Li, Huanjun Chen, Shuxiang Wu

**Affiliations:** ^1^ School of Materials Science and Engineering Sun Yat‐sen University Guangzhou 510275 China; ^2^ State Key Laboratory of Optoelectronic Materials and Technologies Guangdong Province Key Laboratory of Display Material and Technology School of Electronics and Information Technology Sun Yat‐sen University Guangzhou 510275 China; ^3^ State Key Laboratory of Optoelectronic Materials and Technologies Guangzhou Key Laboratory of Flexible Electronic Materials and Wearable Devices School of Materials Science and Engineering Sun Yat‐sen University Guangzhou 510275 China

**Keywords:** broadband photodetector, flexible photodetector, molecular beam epitaxy, sub‐millimeter and Terahertz wave detection, Type‐II Weyl semimetal

## Abstract

Flexible photodetectors with ultra‐broadband sensitivities, fast response, and high responsivity are crucial for wearable applications. Recently, van der Waals (vdW) Weyl semimetals have gained much attention due to their unique electronic band structure, making them an ideal material platform for developing broadband photodetectors from ultraviolet (UV) to the terahertz (THz) regime. However, large‐area synthesis of vdW semimetals on a flexible substrate is still a challenge, limiting their application in flexible devices. In this study, centimeter‐scale type‐II vdW Weyl semimetal, T_d_‐MoTe_2_ films, are grown on a flexible mica substrate by molecular beam epitaxy. A self‐powered and flexible photodetector without an antenna demonstrated an outstanding ability to detect electromagnetic radiation from UV to sub‐millimeter (SMM) wave at room temperature, with a fast response time of ≈20 µs, a responsivity of 0.53 mA W^−1^ (at 2.52 THz), and a noise‐equivalent power (NEP) of 2.65 nW Hz^−0.5^ (at 2.52 THz). The flexible photodetectors are also used to image shielded items with high resolution at 2.52 THz. These results can pave the way for developing flexible and wearable optoelectronic devices using direct‐grown large‐area vdW semimetals.

## Graphical abstract

T_d_‐MoTe_2_ is type‐II Weyl semimetal, which have great potential in THz detection via peculiar topology band structure and exotic transport. T_d_‐MoTe_2_/mica structure grown by MBE is first reported as a photodetector from THz to SMM regime. T_d_‐MoTe_2_/mica detectors show flexible and high‐resolution terahertz imaging, which is suitable for smart wearable devices, contributing to the application of terahertz technology in daily life.

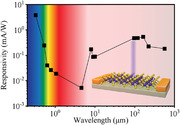

## 1 Introduction

A flexible photodetector is a crucial component for the development of future optoelectronic devices that form the basis of handheld portable devices, bendable high‐speed wireless communications, RF energy harvesting, and wearable electronics.^[^ ^1^, ^2^ ^]^ However, the challenge lies in finding a material system that enables room‐temperature and flexible photodetection with a broadband response covering UV to infrared (IR), especially in the THz and SMM regimes (0.3 THz to 10 THz). Such a system can boost the applications of flexible photodetectors in smart wearable sensors, which are at the heart of the Internet of Things. While traditional semiconductors and quantum structures (e.g., quantum wells and quantum dots) have been widely used in photodetection technology, they usually suffer from narrow detection bands limited by their finite bandgaps.^[^ ^3^, ^4^ ^]^ Additionally, semiconductors (such as HgCdTe and PbSe) used in mid‐infrared (MIR) and THz detectors typically require cryogenic temperatures to suppress thermal noises. The THz detectors will be further integrated with antennae to enhance the coupling with THz radiation. This results in devices that have a large footprint and are complex.^[^ ^5^, ^6^ ^]^ Moreover, conventional semiconductors are typically solid and brittle, which makes them unsuitable for use in mechanically flexible devices.

Due to the exotic optical and optoelectronic responses they grant, two‐dimensional (2D) vdW crystals have garnered much attention in recent years as a new type of material that might revolutionize photodetection technology.^[^ ^7^, ^8^, ^9^, ^10^, ^11^ ^]^ Among the various vdW crystals, Dirac/Weyl semimetals exhibiting linear band structures close to the Dirac/Weyl node, such as WTe_2_,^[^ ^7^ ^]^ PtTe_2_,^[^ ^8^ ^]^ PdTe_2_,^[^ ^9^ ^]^ and NiTe_2_,^[^ ^10^ ^]^ and others, have opened up a new landscape for exploring novel photodetection technologies. First, the gapless band structure can confer the semimetals with a broadband photosensitivity covering the UV to the far‐IR spectral regime. Second, the linear band structure can give rise to massless, relativistic charge carriers with ultrahigh mobilities. Therefore, photodetectors with ultrafast responses and broad operation bandwidths can be envisioned. Last but not least, the atomic thickness of the vdW semimetals can enable tunable optical and electronic properties via electrostatic gating and offer excellent mechanical flexibility, which benefits various flexible and tunable photodetection applications. Accordingly, recent years have witnessed a burgeoning interest in exploring photodetectors using various vdW semimetals, which are superior to conventional photodetectors in terms of room‐temperature broadband photoresponse from UV to THz regime,^[^ ^8^, ^9^, ^10^ ^]^ ultrafast response time,^[^ ^8^, ^9^, ^10^, ^11^ ^]^ as well as polarization‐resolved response.^[^ ^9^, ^10^, ^12^, ^13^ ^]^

The vdW semimetals can generally be categorized into two types, namely type‐I and type‐II semimetals. Type‐II semimetals have strongly tilted Dirac/Weyl cones with open Fermi surfaces, resulting in a large density of states near the Dirac/Weyl node, in contrast to type‐I semimetals where the Fermi surfaces are closed.^[^ ^14^, ^15^ ^]^ This property significantly enhances the electromagnetic absorption and the corresponding response of photodetectors, particularly in the long‐wavelength regime. Moreover, the heavily tilted linear electronic dispersion of type‐II semimetals allows for the development of ultrafast photodetectors that operate in an unbiased self‐powered mode. Despite the development of various broadband photodetectors using type‐II vdW semimetals,^[^ ^7^, ^12^, ^13^, ^16^, ^17^ ^]^ they are mainly reliant on exfoliated vdW flakes with finite lateral size. This limitation severely restricts their application in high‐throughput and large‐area photodetection, such as the development of flexible and wearable photodetectors. To date, direct synthesis of high‐quality and large‐area (with a lateral size over a centimeter scale) type‐II semimetals remains challenging.

Here, we report on the successful growth of large‐scale T_d_‐MoTe_2_ vdW thin films, a type‐II semimetal, using the molecular‐beam epitaxy (MBE) method, with lateral sizes of up to 2.0 × 2.0 cm^2^. Importantly, the films were grown on a flexible mica substrate, enabling the development of a flexible photodetector with ultra‐broadband sensitivity ranging from UV to SMM spectral ranges (325 nm to 566.0 µm). Due to the strong coupling of T_d_‐MoTe_2_ with long wave irradiation, the photodetector does not require an antenna for detecting THz and SMM waves, thus simplifying the device architecture. With its tilted energy band, the photodetector can operate in a self‐driven mode, with a fast response time of ≈20 µs, a responsivity of 0.53 mA W^−1^ (at 2.52 THz), and a NEP of 2.65 nW Hz^−0.5^ (at 2.52 THz). Moreover, the flexible photodetector can achieve high‐resolution THz‐imaging, enabling the detection of shielded items. These results not only reveal the potential application of the semimetal T_d_‐MoTe_2_ in future high‐performance broadband photodetectors but also pave the way for developing flexible and wearable optoelectronic devices using directly grown large‐area vdW crystals.

## 2 Results and Discussion

**Figure 1:**
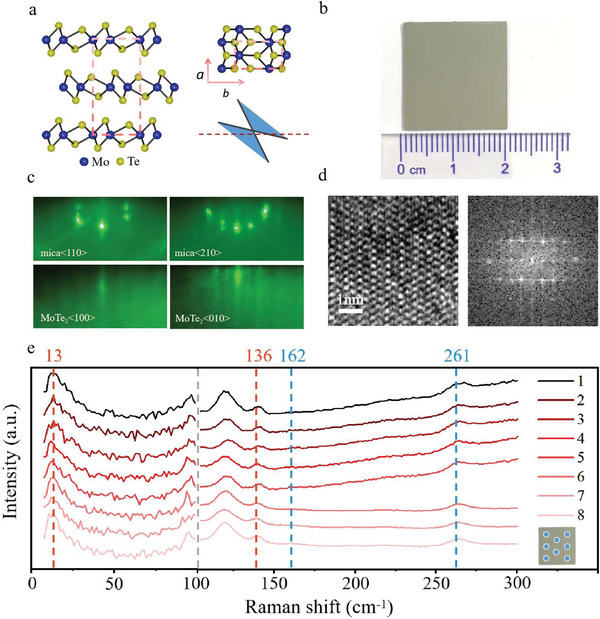
Growth and characterizations of the vdW T_d_‐MoTe_2_ crytal. a) Atomic structure and Weyl point schematics of the T_d_‐MoTe_2_ crystal. b) Optical image of the centimeter‐scale T_d_‐MoTe_2_ thin film. c) RHEED patterns of the mica substrate and T_d_‐MoTe_2_. d) HRTEM images of T_d_‐MoTe_2_ and fast Fourier transform from the layered T_d_‐MoTe_2_. e) The Raman spectrums for T_d_‐MoTe_2_ film show active Raman phonon modes at 13, 133, 161, and 261 cm^−1^ (The white points represent the different Raman measurement locations of the T_d_‐MoTe_2_ thin film).

The T_d_‐phase MoTe_2_ is a type‐II vdW Weyl semimetal with an orthorhombic lattice that breaks inversion symmetry, and it has a tilted three‐dimensional Dirac cone band structure (as shown in **Figure** **1**a).^[^ ^18^, ^19^, ^20^, ^21^, ^22^ ^]^ In our study, we grew epitaxial films of T_d_‐MoTe_2_ on high‐quality hexagonal mica substrate measuring 2.0 × 2.0 cm^2^ using MBE, and the high film uniformity is demonstrated in the optical image presented in Figure 1b. During the growth process, we used reflection high‐energy electron diffraction (RHEED) to monitor the film thickness, crystal quality, and lattice relationship between the film and substrate in situ. After 30 min of growth, we obtained a MoTe_2_ film with a thickness of ≈4 nm (equivalent to 6 monolayers). Figure 1c illustrates the RHEED patterns of mica along the <110> and <210> azimuths, which confirm the hexagonal symmetry of the mica surface. The RHEED patterns of the MoTe_2_ film along <100> and <010> azimuths, shown in Figure 1c, indicate an orthogonal symmetry of the epitaxial film, excluding the formation of the 2H phase. The in‐plane lattice constants of MoTe_2_ were calculated to be *a* = 3.73 Å and *b* = 6.73 Å. Additionally, the HRTEM image and regular spot configuration in fast Fourier transform, as shown in Figure 1d, confirm the orthogonal symmetry of the MoTe_2_ surface. To differentiate between the 1T′‐phase (monoclinic P21/m) and T_d_‐phase (orthorhombic Pmn21), Raman measurements were conducted at room temperature. The spectra between 150 and 300 cm^−1^ reveal two dominant modes at 162 cm^−1^ and 261 cm^−1^ (blue lines), which are significantly different from those of the 2H phase, indicating the presence of T_d_ phase.^[^ ^25^, ^26^, ^27^, ^28^, ^29^ ^]^ Moreover, the shear mode at 13 cm^−1^ and the out‐of‐plane vibration at 136 cm^−1^ (red lines) further confirm the T_d_ phase of the epitaxial MoTe_2_ on mica.^[^ ^30^, ^31^, ^32^ ^]^ Raman spectroscopy measurements taken from different locations in the same film confirm that the samples are T_d_ phases of MoTe_2_ grown on mica substrates via MBE and illustrate great film uniformity in centimeter scale (Figure 1e). To further demonstrate the repeatability of the experiment, Raman measurements were carried out for several samples grown in the same way but at different source powers (Figure S1 in Supporting Information), showing the repeatability of T_d_‐MoTe_2_ samples.

**Figure 2:**
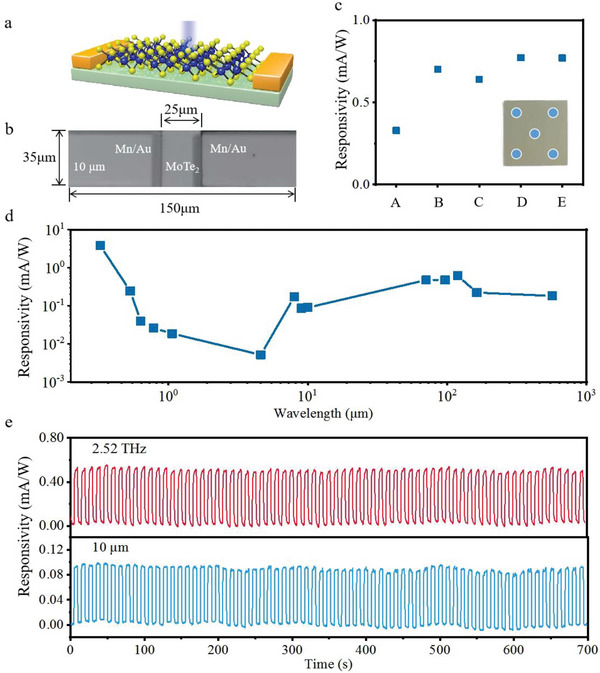
Fabrication and characterization of the T_d_‐MoTe_2_/mica photodetector. a) Schematic showing the photodetector of T_d_‐MoTe_2_/mica. b) Optical microscope image of the T_d_‐MoTe_2_/mica photodetector with a channel length of 25 µm, a channel width of 35 µm, and the active area is 150 × 35 µm^2^. c) Responsivity of the T_d_‐MoTe_2_ photodetectors fabricated at different locations inside a large T_d_‐MoTe_2_ thin film. The bias voltage is 0.1 V, the illumination frequency is 2.52 THz. The white points represent the different photodetectors. d) Wavelength‐dependent responsivity of the T_d_‐MoTe_2_/mica photodetector, showing an ultrabroadband response with responsitivies of 3798.0, 245.4, 40.0, 26.3, 18.7, 5.2, 171.6, 86.7, 88.3, 475.3, 477.2, 533.3, 222.7, and 182.0 µA/W at 325 nm, 532 nm, 633 nm, 785 nm, 1064 nm, 4.6 µm, 8 µm, 9 µm, 10 µm, 4.24 THz (70.8 µm), 3.11 THz (96.5 µm), 2.52 THz (119.0 µm), 1.84 THz (163.0 µm), and 0.53 THz (566.0 µm), respectively. The bias voltage is 0.1 V.

The Metal‐Semimetal‐Metal structure of the T_d_‐MoTe_2_/mica photodetector was fabricated using MBE and masking technology to characterize its spectral response. The schematic and micrographs of the device are shown in **Figure** **2**a,b, respectively, indicating that the device dimensions are 150 µm in length and 35 µm in width. The detector's performance was demonstrated at room temperature using radiation with wavelengths of 325 nm, 532 nm, 633 nm, 785 nm, 1064 nm, 4.6 µm, 8 µm, 9 µm, 10 µm, 4.24 THz (70.8 µm), 3.11 THz (96.5 µm), 2.52 THz (119.0 µm), 1.84 THz (163.0 µm), and 0.53 THz (566.0 µm) and active powers ranging from 0.008 mW to 5.7 mW. The active power was calculated from the photoactive area of the detector, which is shown in Figure 2b and has an area of 150 × 35 µm^2^. Here, the responsivities under irradiation from the UV to THz and SMM regimes are calculated by the formula: $R=\frac{I_{light}-I_{\mathrm{dark}}}{P_{\mathrm{light}}}$, where *I*_light_ is the current under irradiation, *I*_dark_ is the dark current, and *P*_light_ is active power of lasers. Theoretically, the photoresponse experiment on T_d_‐MoTe_2_ devices should be extended to include wavelengths with lower photon energies, due to the gapless linear dispersion in the type‐II vdW Weyl semimetal band structure.^[^ ^18^, ^19^, ^20^, ^33^, ^34^ ^]^ Therefore, different devices were fabricated at various locations on the same film for terahertz detection, as shown in Figure 2c. The devices exhibited approximate THz responses with responsivity values of 0.53, 0.56, 0.77, 0.70, and 0.33 mA W^−1^, respectively. The terahertz responsivity in Figure 2c, along with the optical image in Figure 1b and the Raman spectroscopies in Figure 1e, collectively demonstrate the uniformity of the T_d_‐MoTe_2_ film and its optoelectronic properties across different locations.

The T_d_‐MoTe_2_ device can be classified into UV, visible, IR, THz, and SMM detectors based on the wavelength (or frequency) of irradiation, as demonstrated by the wavelength‐dependent responsivity in Figure 2d. Moreover, the stable responsivities and noise levels of the seventy cycles in the MIR‐SMM regime suggest the reliable photodetection of T_d_‐MoTe_2_/mica architecture (Figure 2e). The maximum responsivity was achieved at 325 nm, with a value of 3.80 mA W^−1^. In the MIR regime, a value of 171.6 µA W^−1^ can be obtained (8 µm). Notably, a responsivity of 533.3 µA W^−1^ at 2.52 THz (119.0 µm) was obtained in the absence of a THz antenna, simplifying micromachining and facilitating the fabrication of flexible integrated sensor arrays. In addition, the current−voltage curve in the UV−SMM regime (Figure S2 in Supporting Information) reflects good Ohmic contact and linear dependence under a bias voltage.

**Figure 3:**
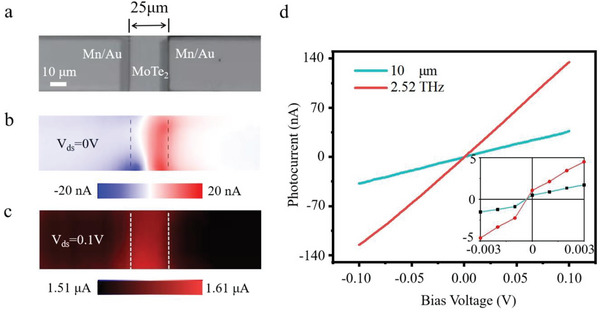
Scanning photocurrent microscopy characterizations of the T_d_‐MoTe_2_/mica photodetector. a) Optical microscope image of the T_d_‐MoTe_2_/mica photodetector with a channel length of 25 µm and a channel width of 35 µm. b,c) Scanning photocurrent microscopy images at 10 µm under bias voltage of (b) 0 V and (c) 0.1 V, respectively. The power of the MIR laser is ≈9 mW, with a focused spot of 21 µm in diameter. d) Bias voltage‐dependent photocurrent at 10 µm and 2.52 THz. Inset: enlarged photocurrent generation versus bias voltage.

To reveal the broadband photoresponse mechanism of T_d_‐MoTe_2_/mica photodetector, the photocurrent distribution in the device area (≈150 × 35 µm^2^), including the channel and the overlap region of metal‐MoTe_2_ (**Figure** **3**a), was imaged via scanning photocurrent microscopy at 10.0 µm. The light source used was a laser with a power of 9 mW, focused on a diameter of 21 µm. In situ scanning photocurrent images were captured under zero and 0.1 V bias and are shown in Figure 3b,c, respectively.

Under irradiation without any bias voltage, a positive photoresponse was observed at a distance of 10 µm away from the electrodes, while the opposite photocurrents were generated at the interface of MoTe_2_‐metal junctions, as depicted in Figure 3b. In addition, nonzero photocurrents were also observed in the bias‐dependent photocurrent experiment at 10.0 µm and 2.52 THz (Figure 3d). This phenomenon can be attributed to the photothermoelectric effect (PTE), which is caused by the electron temperature gradient resulting from differential thermoelectric power and optical excitation.^[^ ^35^, ^36^, ^37^ ^]^ In gapless semimetals, such as T_d_‐MoTe_2_, the Shockley–Ramo mechanism leads to the photocurrent generated by PTE being independent of the proximity of the metal‐MoTe_2_ junction.^[^ ^38^ ^]^ Furthermore, in type‐II vdW Weyl semimetal T_d_‐MoTe_2_, the strongly tilted Weyl cone induces asymmetric excitation of carriers under irradiation, resulting in the self‐powered photocurrent.^[^ ^39^ ^]^ Therefore, both PTE and the asymmetric excitation of carriers by the tilted Weyl cone consistently generate the photocurrent under 0 V bias.

**Figure 4:**
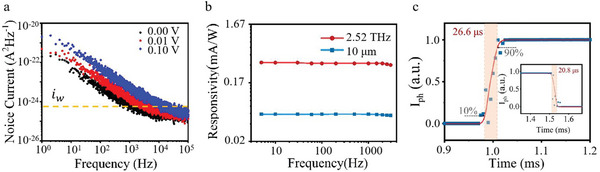
Frequency response and noise characteristics of the T_d_‐MoTe_2_/mica photodetector. a) The current noise power spectrum measured from 1 Hz to 100 kHz. It is applicable for the spectral regime from UV to SMM. The white noise was calculated as shown in the Supporting Information. b) Frequency‐dependent photoresponse at 2.52 THz and 10.0 µm. c) Photoresponse speed of a typical T_d_‐MoTe_2_/mica photodetector under 2.52 THz illumination.

When subjected to a 0.1 V bias, the photocurrent exhibited linear amplification, as depicted in Figure 3d. Based on the distribution of the positive photocurrent, it was observed that the photocurrent in the center of the channel was significantly higher than in the contact region. The detector's performance was assessed using current noise and noise equivalent power. The total noise current (*i*_N_) was composed of 1/*f* noise and white noise (*i*_w_), with 1/*f* noise dominating the noise current at 1 Hz, as shown in **Figure** **4**a (white noise is elaborated in the Supporting Information). NEP corresponds to the minimum detectable power with a 1 Hz bandwidth, which is extracted from *NEP* = *i*_N_/*R*_A_, where *i*_N_ is the total noise current, and *R*_A_ is the responsivity. Based on the calculated *R*_A_ and NEP values, it was demonstrated that the T_d_‐MoTe_2_/mica detector had a responsivity of 88.3 µA W^−1^ and an NEP value of 16.0 nW Hz^−0.5^ at 10.0 µm. Furthermore, a responsivity of 533.3 µA W^−1^ and an NEP value of 2.65 nW Hz^−0.5^ at 2.52 THz (119.0 µm) without THz antenna were achieved, which reduced the complexity of micromachining and provided a relatively brief premise for the fabrication of flexible integrated sensor arrays.

Modulation frequency‐dependent photoresponse measurements were conducted at 10.0 µm and 2.52 THz irradiation under 0.1 V bias to demonstrate the speed of photodetection. Figure 4b shows that at a modulation frequency of only 5 Hz, the responsivity was reduced by 34% and 32% at both THz and MIR regimes compared to continuous irradiation (Figure 2e), respectively. In contrast, the remaining responsivity decreased by less than 10% (4.7% at 10.0 µm, 9.2% at 2.52 THz) as the modulation frequency was increased to 3000 Hz. This illustrates that the photocurrent at THz and MIR under 0.1 V bias contains two main mechanisms with different response speeds. Additionally, the accurate response speed of 26.6/20.8 µs (rise/fall time) at 2.52 THz was determined from a single magnified photoresponse curve, which was obtained directly from the high‐speed sampling oscilloscope (Figure 4c and Figure S3 in Supporting Information). First, the extremely slow photocurrent response generated by the photobolometric effect (PBE)^[^ ^35^, ^40^, ^41^ ^]^ is caused by the uniform heating effect resulting from photon absorption in the MIR to THz range, leading to the generation of hot carriers and alteration of MoTe_2_ conductivity. This in turn generates photocurrent when a bias voltage is applied. Typically, the slow photocurrent response can be filtered out at high chopping frequencies and does not affect the actual THz imaging speed. On the other hand, the fast photoresponse in our detector (faster than 333 µs) is generated by the photoelectric effect of gapless dispersion in three‐dimensional momentum space. This effect enables low‐energy photons to excite carrier transitions and is critical to the performance of detectors based on other semimetals.^[^ ^35^ ^]^ Additionally, in the absence of bias, the electron−hole pairs compound rapidly, resulting in increased responsivity by breaking symmetry. Overall, both PBE and photoelectric effect based on type‐II Weyl cone consistently generate photocurrent under bias voltage.

**Figure 5:**
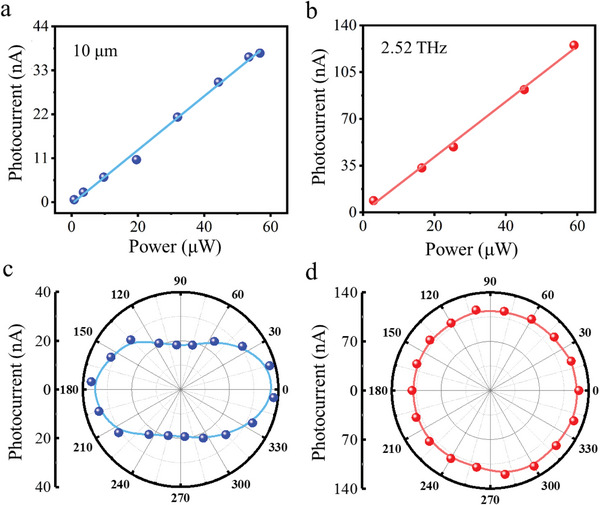
Dependent of photocurrent of the T_d_‐MoTe_2_/mica photodetector on the illumination power and polarization. a,b) Power‐dependent photocurrent of the detector under 10.0 µm and (b) 2.52 THz illuminations, respectively. c,d) Dependence of the photocurrent on the excitation polarization, with an illumination frequency of 10.0 µm (c) and 2.52 THz (d), respectively. The ratios of the polarization anisotropy ire 1.95 (c), and 1.12 (d), respectively.

To investigate the behavior of the T_d_‐MoTe_2_/mica detector under different laser powers at THz and MIR, we measured the power dependence of the photocurrent under 0.1 V bias, as shown in **Figure** **5**a,b. The photocurrent exhibited linear dependence on the light power in both the MIR and THz bands, which confirms the mechanism as the free electron absorption of the Dirac fermions.^[^ ^8^, ^42^, ^43^ ^]^ Moreover, the excellent linear property facilitates the fabrication of optical imaging devices. To gain further insight into the performance of T_d_‐MoTe_2_/mica, we measured the polarization‐dependent photocurrent response under 0.1 V bias at 10.0 µm and 2.52 THz. The polarization states of the THz and MIR sources were fixed using a linear polarizer, and the device was rotated with respect to the allowing direction of the polarizer during the measurements. As illustrated in Figure 5c,d, the photodetector exhibited an anisotropic response to polarized irradiation at 10.0 µm, with the maximum photocurrent response along the a‐axis of the T_d_‐MoTe_2_ crystal. These results are consistent with previous studies on type‐II semimetals,^[^ ^9^, ^10^, ^12^, ^13^ ^]^ showing that the strongly tilted gapless band structure can lead to anisotropy of electromagnetic waves in the type‐II vdW Weyl semimetal T_d_‐MoTe_2_, thus causing the anisotropic photocurrent.

Moreover, Figure 2d illustrates that the photocurrent is higher in the THz band than in the MIR band. This may be due to the Fermi level approaching the Weyl point (only 5–6 meV),^[^ ^18^, ^20^ ^]^ indicating that carriers are excited near the Fermi level when the photon frequency is close to ≈3 THz. This increases the contribution of photon absorption to the photocurrent. Additionally, under the same power density of irradiation, the increasing number of low‐energy photons leads to an increase in the photoexcited carriers at THz, similar to Weyl semimetal TaAs^[^ ^12^ ^]^ and Dirac semimetal PtTe_2_^[^ ^13^ ^]^ at higher frequencies of irradiation. When photon energy is further reduced, the contribution of the photocurrent will transform from the interband transition to the intraband transition.^[^ ^44^, ^45^, ^46^, ^47^, ^48^ ^]^ Due to the Pauli blocking, the photocurrent will be suppressed and constrained.

**Figure 6:**
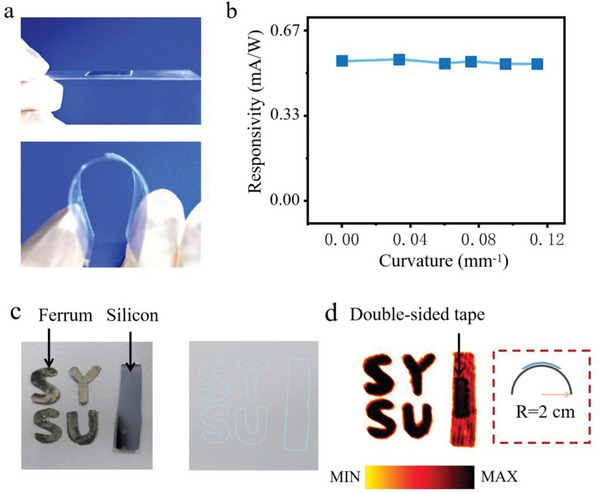
THz imaging using the flexible T_d_‐MoTe_2_/mica photodetector. a) Photographs of the flexible T_d_‐MoTe_2_/mica photodetector. b) Relationship between the photodetector bending curvature and responsivity under 2.52 THz irradiation. The data points from left to right represent ∞, 29.3, 16.6, 13.25, 10.47, and 8.8 mm radius of curvature, respectively. c) Photographs of Ferrum lettering and Silicon stuck on the paper with 3 M double‐sided tape. d) 2D scanning imaging of Ferrum lettering and Silicon stuck using the bending T_d_‐MoTe_2_/mica detector, with a bending curvature radius of 2 cm.

To demonstrate its flexibility, the detector was bent to different bending radii to characterize the photocurrent at 2.52 THz (**Figure** **6**a). As shown in Figure 6b, the T_d_‐MoTe_2_/mica device exhibited stable THz detection performance when bent to a curvature radius of 8.8 mm, indicating that the device is suitable for different parts of the human body's surface structures. The choice of mica and MBE technology consistently realized flexible THz sensors. Next, THz imaging were carried out by raster scanning the detector upon 2.52 THz illumination to verify the practical application of the T_d_‐MoTe_2_/mica detector (Figure 6c). A 2D scanning with a speed of 1 mm s^−1^ was performed to image shielded items (ferrous letters and a silicon wafer stick with paper) under 0.1 V bias. As shown in Figure 6d (bent situation) and Figure S7, Supporting Information, (flat situation), images with 107 × 107 pixels were obtained, which can identify the shape features of ferrum and silicon, and distinguish ferrous and silicon materials by their color differences. The tape signal can be even identified on the back of silicon. From the comparison shown in **Table** **1**, it can be seen that although our device's various performance indicators are not significantly better than other topological semimetal‐based devices, its flexible characteristics and the fact that the material can be prepared in large areas give the device a significant advantage in wearable optoelectronic applications.

**Table 1:** Summary of topological semimetals used for novel photodetectors

Materials	Wavelength range	Flexible [YES or NO]	Fabrication (Large scale [YES or NO])	Self‐power [YES or NO]	THz Antenna [YES or NO]	THz Responsivity	THz NEP [nW Hz^−0.5^] (Bandwidth Hz)	THz tr/td [µs]	Refs.
Graphene (SLG)	0.3 THz	NO	Mechanical exfoliation(NO)	NO	YES	100 mV W^−1^	≈200	‐	[49]
(BLG)						150 mV W^−1^	≈30	‐	
Graphene ‐ quartz	0.5 THz to 1.6 THz	NO	Mechanical exfoliation(NO)	NO	NO	‐	‐	100	[50]
ZrGeSe	0.1 THz to 0.27 THz	NO	PVT(YES)	YES	YES	110 mA W^−1^	0.15 (>1000 Hz)	8.1 /6.3	[51]
PtTe_2_	650 nm to 10.7 µm	NO	MBE(YES)	NO	‐	‐	‐	‐	[13]
PtTe_2_	0.04 THz to 0.3 THz	NO	CVD(YES)	Yes	YES	30–250 mA W^−1^	0.042–0.149 (1 Hz)	7/8	[8]
PdSe_2_	200 nm to 3044 nm	NO	Selenization process (YES)	YES	‐	‐	‐	‐	[52]
PtSe_2_	360 nm to 2000 nm	NO	CVD(YES)	YES	‐	‐	‐	‐	[53]
InSe	470 nm to 980 nm	NO	Mechanical exfoliation(NO)	NO	‐	‐	‐	‐	[54]
PdTe_2_	0.04 THz to 0.3 THz	NO	Mechanical exfoliation(NO)	YES	YES	10 000 mA W^−1^	0.001–0.05 (1 Hz)	1/2.2	[9]
NiTe_2_	0.04 THz to 0.3 THz	NO	Mechanical exfoliation(NO)	YES	YES	250–12570 mA W^−1^	0.0049– 0.0898 (1 Hz)	2.7	[10]
MoSe_2_	0.29 THz	NO	CVD(YES)	NO	NO	38 mV W^−1^	6.6 × 10^6^	‐	[55]
Cd_3_As_2_	532 nm to 10.6 µm	NO	CVD(YES)	YES	NO	‐	‐	‐	[16]
Cd_3_As_2_	0.04 THz to 0.3 THz	NO	MBE(YES)	NO	NO	40 mA W^−1^	0.43 (1 Hz)	15/14	[11]
ZrTe_5_	532 nm to 632 nm	NO	Mechanical exfoliation(NO)	YES	‐	‐	‐	‐	[56]
TaIrTe_4_	532 nm to 10.6 µm	NO	Mechanical exfoliation(NO)	YES	‐	‐	‐	‐	[17]
WTe_2_	514 nm to 10.6 µm	NO	Mechanical exfoliation(NO)	NO	‐	‐	‐	‐	[7]
TaAs	438 nm to 10.3 µm	NO	PVT(YES)	YES	‐	‐	‐	‐	[12]
T_d_‐MoTe_2_/mica	325 nm to 0.53 THz	YES	MBE(YES)	YES	NO	0.53 mA W^−1^	2.7 (1 Hz)	26.6 /20.8	This work

## 3 Conclusions

In summary, we have developed a MBE‐based method for growing centimeter‐scale type‐II vdW Weyl semimetal T_d_‐MoTe_2_ on a flexible mica substrate. On the basis of such large‐area semimetal, we developed a flexible photodetector with an ultra‐broadband photocurrent response from the UV to SMM regime (325 nm – 566.0 µm), either with bias voltage or operated in a self‐powered mode. The photodetection mechanism of the device can be ascribed to a combination of PTE, PBE, and photoelectric effect near the type‐II Weyl cone. We further employed such device for THz imaging of shielded items with a high resolution at 2.52 THz. We believe that these results will be important for developing of flexible and wearable optoelectronic devices in the MIR and THz spectral regions.

## 4 Experimental Section

### Growth Details and Characterization

T_d_‐MoTe_2_ films were deposited onto mica substrate using MBE system from OMICRON. The substrate was maintained at a temperature of 255 °C during deposition. High purity Te (99.99999%) was evaporated at 330 °C from an exuding cell, while Mo rod (99.95%) was evaporated using an e‐beam evaporator, resulting in a growth rate of ≈5 min ML^−1^ (with a thickness of 6 ML for the obtained large‐area T_d_‐MoTe_2_ film). RHEED was employed to ensure a high surface quality, and further characterization was performed using a high‐resolution transmission electron microscope (HRTED: Titan G2 60–300). For the HRTEM experiment, samples were grown on a few‐layer graphene transferred onto a Mo net prior to film growth. Raman measurements were carried out using a commercial Raman equipment (Renishaw inVia Reflex) with 532 nm (0–100 cm^−1^) and 514.5 nm (100–300 cm^−1^) lasers.

### Device Fabrication

To prevent oxidation of the T_d_‐MoTe_2_ film, an Al thin film was deposited on top of it and then oxidized in ambient atmosphere to serve as a capping layer. Au metal electrodes were then fabricated using MBE and masking technology to create channels with a length of 25 µm and a width of 35 µm.

### Photoresponse Measurements

All measurements were conducted at room temperature. The electrical and photocurrent characteristics were measured using a Keithley 2636 B instrument. The light sources used in the study comprised semiconductor lasers (325 nm, 532 nm, 633 nm, 785 nm, and 1064 nm), quantum cascade lasers (QD4500CM1, Thorlabs: 4.6 µm; MIRCat S/N10016, Daylight: 8.0 to 11.0 µm), and THz lasers (FIRL 100, Edinburgh Instruments: 4.24 THz, 3.11 THz, 2.52 THz, 1.84 THz, and 0.53 THz). To control the chopping frequency in the frequency‐dependent photocurrent response measurements, a lock‐in amplifier (Stanford SR830) was used. A linear polarizer was employed for fixing the polarization state of the incident electromagnetic waves. Response time of the photodetector was obtained directly from a high‐speed sampling oscilloscope (Tektronix DPO 7354C).

## Conflict of Interest

The authors declare no conflict of interest.

## Supporting information

Supporting Information

## Data Availability

The data that support the findings of this study are available from the corresponding author upon reasonable request.
