# Supplementary material for: A Centimeter‐Scale Type‐II Weyl Semimetal for Flexible and Fast Ultra‐Broadband Photodetection from Ultraviolet to Sub‐Millimeter Wave Regime
Source: Adv Sci (Weinh). 2023 Apr 24;10(17):2205609. doi: 10.1002/advs.202205609 (PMC10265072; doi:10.1002/advs.202205609)
Supplement: Supplementary file 1 — Supporting Information [file ADVS-10-2205609-s001.pdf]

ADVANCED SCIENCE
Open Access

Supporting Information

for *Adv. Sci.*, DOI 10.1002/advs.202205609

A Centimeter-Scale Type-II Weyl Semimetal for Flexible and Fast Ultra-Broadband Photodetection from Ultraviolet to Sub-Millimeter Wave Regime

Qi Yang, Ximiao Wang, Zhihao He, Yijun Chen, Shuwei Li, Huanjun Chen and Shuxiang Wu**

Supporting Information

A Centimeter-Scale Type-II Weyl Semimetal for Flexible and Fast Ultra-Broadband Photodetection from Ultraviolet to Sub-Millimeter Wave Regime

Qi Yang†, Ximiao Wang†, Zhihao He†, Yijun Chen, Shuwei Li, Huanjun Chen, and Shuxiang Wu**

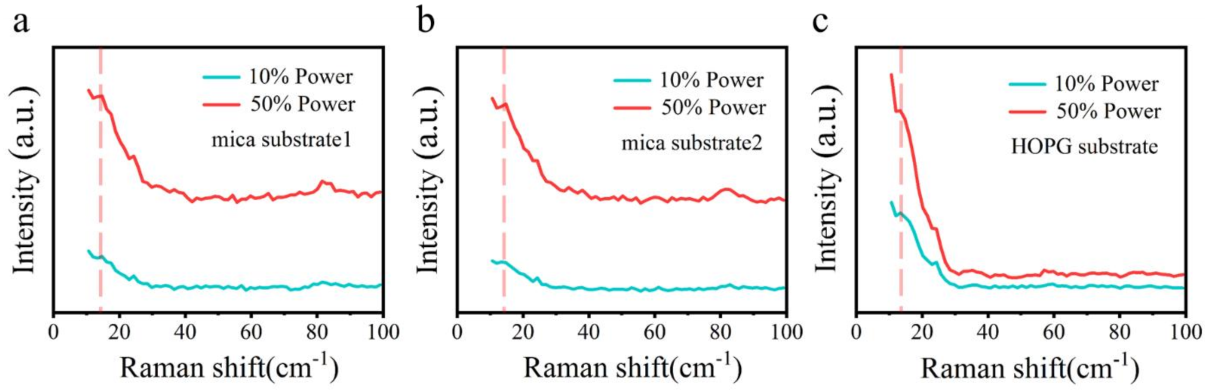

Figure S1. Raman spectrums for different T_d-$MoTe_2$ samples under the same growth conditions.

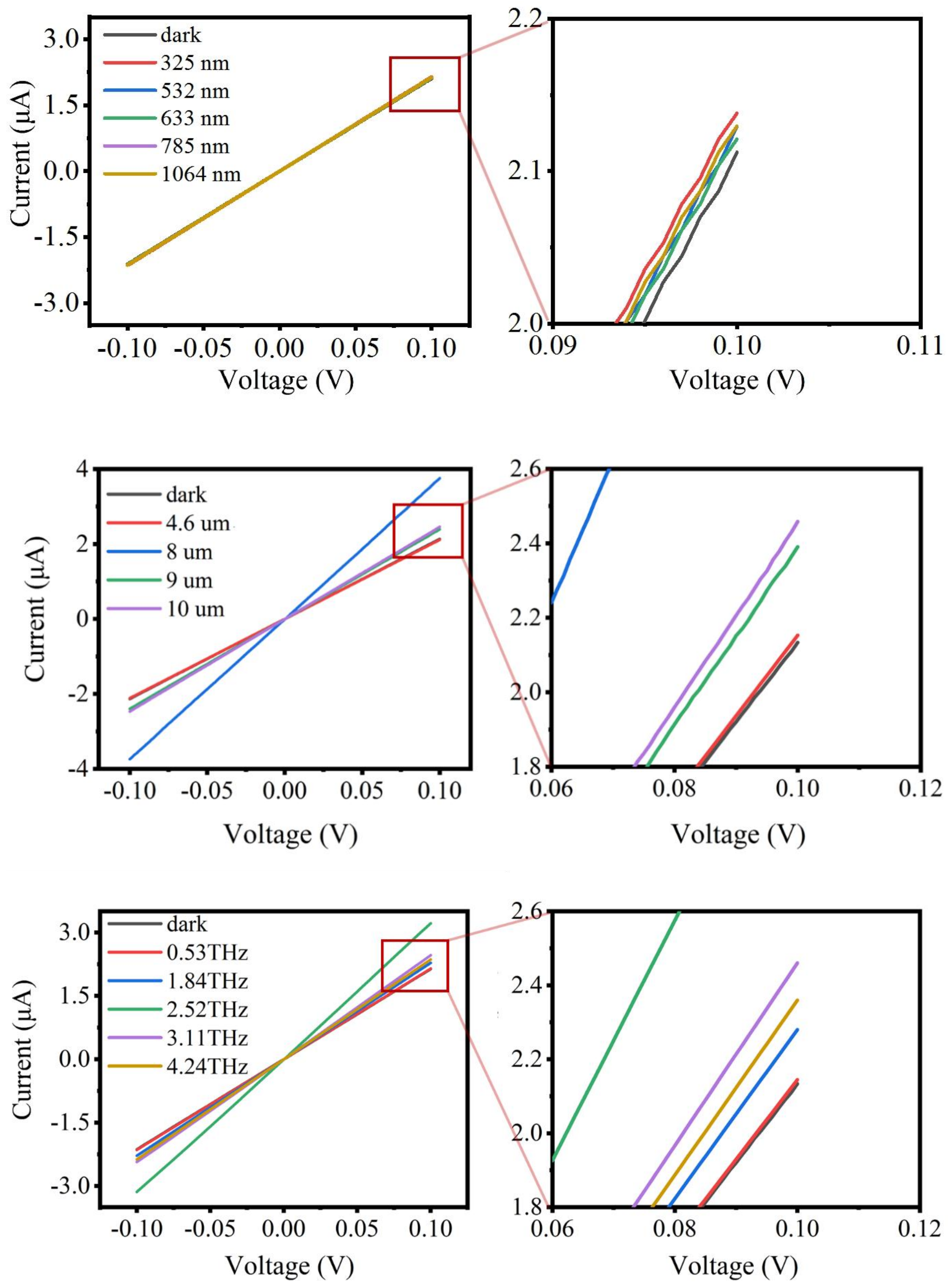

Figure S2. Current-Voltage curve at Visible, MIR, THz and SMM regimes.

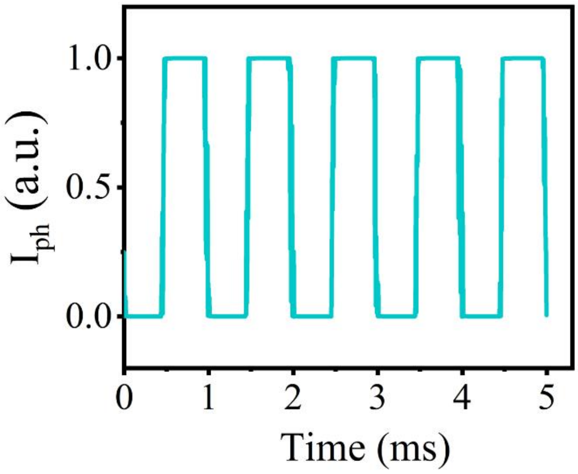

Figure S3. Photoresponse at 2.52 THz were obtained directly from the high-speed sampling oscilloscope.

Comparison of noise

The current generated by 0.1 V bias applied during the operation of T_d-$MoTe_2$/mica photodetector introduces shot noise, which cannot be avoided. Shot noise is obtained by the following expression:

$$i_s = \sqrt{2qI_dB} \qquad (Equation\ S4)$$

where q is the electron charge; I_d is the dark current at 0.1 V bias; B is the bandwidth, 1 Hz. Meanwhile, the thermal noise caused by random thermal motion of electrons is also a non-negligible component of white noise, and can be calculated by the following expression:

$$\mathrm{i_t} = \sqrt{\frac{\mathrm{4k_BTB}}{\mathrm{R}}} \qquad (Equation\ S5)$$

where k_B is the Boltzmann constant; T is the thermodynamic temperature, 300 K; R is the resistance of device. Therefore, the total white noise is calculated by the following expression:

$$i_w = \sqrt{i_s^2 + i_t^2} \qquad (Equation\ S6)$$

and it is 9750 $pA\cdot Hz^{-1/2}$.

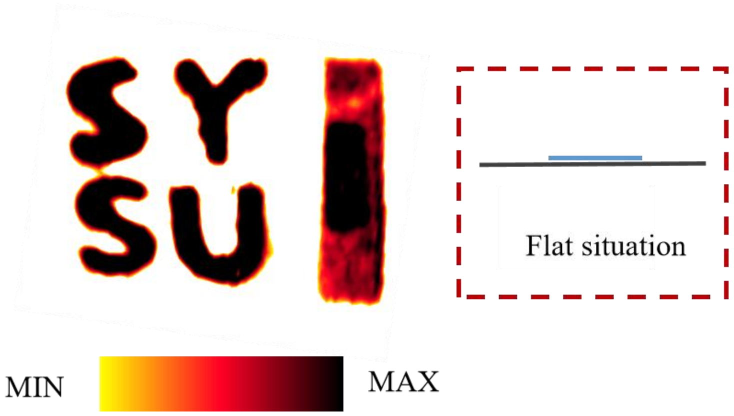

Figure S7. 2D scanning image without bending.
